# Chemotherapeutic nanoparticles for glioblastoma

**DOI:** 10.3389/fonc.2025.1641752

**Published:** 2025-08-11

**Authors:** Samantha Messina, Candida Zuchegna, Mara Bruzzi

**Affiliations:** ^1^ Dipartimento di Scienze, Università di Roma Tre, Roma, Italy; ^2^ Department of Epidemiology, Preclinical Research and Advanced Diagnostics, National Institute for Infectious Diseases Istituto di Ricovero e Cura a Carattere Scientifico (IRCCS) ‘L. Spallanzani’, Rome, Italy; ^3^ Dipartimento di Fisica e Astronomia, Università degli Studi di Firenze, Sesto Fiorentino, Italy; ^4^ Istituto Nazionale di Fisica Nucleare-Istituto Nazionale di Fisica Nucleare (INFN), Sezione di Firenze, Sesto Fiorentino, Italy

**Keywords:** nanoparticles, delivery, glioblastoma, chemotherapy, radiotherapy, PDT

## Abstract

Therapeutic agents into the brain are a major challenge for treatment of brain cancer due to the blood-brain barrier (BBB) that prevents many drugs from reaching the brain. The deadliest form of brain cancer is glioblastoma (GBM), and its current standard treatment involves surgical removal of the tumor, followed by chemotherapy and radiotherapy. The main limitations of chemotherapy for brain tumors are BBB permeability, lack of specificity, and potential damage to healthy tissue. Enhanced molecular understanding of the underlying glioblastoma pathogenesis doesn’t lead to better therapeutic options. The emergence of nanotechnologies offers a promising solution, as controlled drug delivery using nanoparticles to bypass the BBB. Nanoparticles embrace a wide range of synthetic and natural biological materials effective in enhancing diagnostic and therapeutic efforts, alone or in combination with immunological, genetic, or cellular therapies. Lipid-based, inorganic, and polymeric nanoparticles are on the cutting edge of precision medicine for cancer as both therapeutic and diagnostic tools. Currently, there is no consensus on the most effective nanoparticle formulation for treating brain tumors, including their size, composition, targeting, and drug delivery mechanisms. Nanoparticles also have some drawbacks, including uncertain toxicity, reproducibility, and high cost. This short review provides a selection of primary research on nanoparticles as delivery chemotherapeutic systems, with a highlight on Photodynamic therapy (PDT) and radiotherapy (RT) combinatorial modalities. Here we critically examine the most significant research findings in the field of nanomedicine as applied to glioblastoma therapy, with a particular emphasis on chemotherapeutic nanoparticle (NP)-based drug delivery. In parallel, we provide an overview of the physicochemical properties of nanoparticles, informed by recent advances in their engineering, with a special focus on combinatorial strategies involving photodynamic therapy (PDT) and radiotherapy (RT). Our analysis focuses on highly potent anticancer drugs that are well characterized in terms of their pharmacokinetics and pharmacodynamics. The latest developments in immunotherapy and molecular-targeted treatments are intentionally excluded. Our viewpoint is grounded in the conventional yet highly effective chemotherapy-based delivery approach, which remains widely used against many of the most lethal human cancers. Despite being underrepresented in current literature, this strategy holds strong potential for clinical translation and competitiveness.

## 1 Introduction

Improved treatments for brain cancer remain an urgent unmet need. Mostly, glioblastoma current therapy includes surgical resection followed by radiation and/or chemotherapy with temozolomide (TMZ) (as a first line treatment) with overall survival times among the worst of any cancer (1–3). Chemotherapy of brain tumors has been mainly limited by a lack of effective methods of drug delivery, due to the blood-brain barrier (BBB) that prevents many drugs from reaching the cancer mass (4, 5). Intratumoral heterogeneity is a hallmark of the disease, showing multiple driving mutations within a single tumor (6) which is reflected in morphological, transcriptional, genetic, epigenetic, functional diversity. This pronounced molecular heterogeneity of glioblastoma hampers advances in the development of chemotherapeutic drugs in comparison with other cancer types. Common glioblastoma driver mutations are PTEN loss, mutant activated epidermal growth factor receptor variant III (EGFR vIII), p53 loss, and overexpression of platelet-derived growth factor receptor A (PDGFR), and, more rarely, activating mutations in B-RAF (7). Despite the deep molecular and histological characterization of glioblastoma based on the current WHO 2021 classification (3), the failure of novel targeted therapies mirrors the complexity of the regulatory network (8, 9), and lastly glioblastoma remains largely elusive to current immunotherapies (10).

Nanotechnology-based therapies involve delivering therapeutic cargo directly to tumor cells. The limited progress made in treating brain tumors is partly due to the inaccuracy of preclinical models. Many types of NPs have been developed such lipid, inorganic and polymeric NPs and many types of therapeutic cargoes ranging from classical alkylating agent such as temozolomide (TMZ), doxorubicin (DOX), cisplatin (CisPt), paclitaxel (PTX) to newest molecular target. Nanoparticle transport across the blood–brain barrier is achieved through two mechanisms: passive accumulation of plain nanocarriers or active targeting of the BBB *via* ligands (such as protein, peptide, aptamer, folate carbohydrates) detectable by receptors located on the BBB and/or glioma membranes. The shortcomings of using nanoparticles relies on poor stability, poor biocompatibility, low tumor retention, and suboptimal drug release control. Moreover, the structural complexity of nanoparticles and the limitations of current methods for nanoparticle physico-chemical characterization are challenging due to parameters such as size, morphology, charge, purity, drug encapsulation and coating efficiency, and density of conjugated ligands. A detailed discussion of the various novel diagnostic and therapeutic approaches to glioblastoma currently being investigated by NPs is beyond the scope of this article. Moreover, we neglect liposomal encapsulation technology showing limited physico-chemical stability due to fragile phospholipid membranes and their peroxidation (11). Herein, we highlight the current state and emerging research directions for pre-clinical studies in nanoparticle approaching clinical applications in glioblastoma chemotherapy, with a special focus on classical alkylating agent cargoes, considering of state-of-art mechanisms and stimuli-responsive strategies enhancing drug delivery.

## 2 Nanotechnologies for glioblastoma

### 2.1 Generation of nanoparticles for glioblastoma

In the brain cancer context, challenges remain in the clinical translation of engineered nanoparticles (NPs) able to cross the BBB. These nanoparticles possess specific intrinsic—such as electronic, optical, and magnetic features—and extrinsic properties —like size, surface-to-volume ratio, or surface energy—to enhance delivery efficiency, minimize off-target effects, and optimize drug kinetics. Acting as “Trojan horses,” they facilitate the delivery of both classical alkylating agents or new biological targeted molecules (i.e. VEGF, antibodies, RNA, and peptides) straight to cancer cells.

Nanoparticle surfaces can be functionalized with targeted ligands capable of selectively binding to receptors expressed on brain endothelial cells, thereby promoting their translocation across the blood–brain barrier *via* mechanisms such as receptor-mediated transcytosis. This is the case of transferrin receptor (TfR) and low-density lipoprotein receptor (LRP1) which serve as common molecular targets on brain endothelial cells exploited by nanoparticles to enable transcytosis (12, 13). Some authors employed glycosylated micelles to deliver bioactive compounds *via* glucose transporter-1 (GLUT1), a key mediator of cerebral glucose uptake. By precisely controlling the glucose density on the nanoparticle surface, they were able to modulate its biodistribution within the brain, thereby significantly enhancing nanocarrier accumulation in cerebral tissue (14). Furthermore, PEGylation lengthened their circulation time in the bloodstream, reducing protein interactions and enhancing their therapeutic efficacy (15). The evolution of nanotechnological systems for glioblastoma therapy is generally classified into different generations, as summarized in **Table 1**.

**Table 1:** Generations of NPs for GBM, key features and pros/cons.

Generation	Key feature	Advantages	Drawback
First	Passive targeting EPR effect	Simple design, enhanced drug stability	Non-specificity, limited BBB crossing
Second	Active targeting ligands, PEGylation	Targeted delivery, reduced toxicity	Immune response, higher complexity
Third	Stimuli-responsive, theranostic	Precision delivery, imaging-guided therapy	High cost, complex production

**Figure 1:**
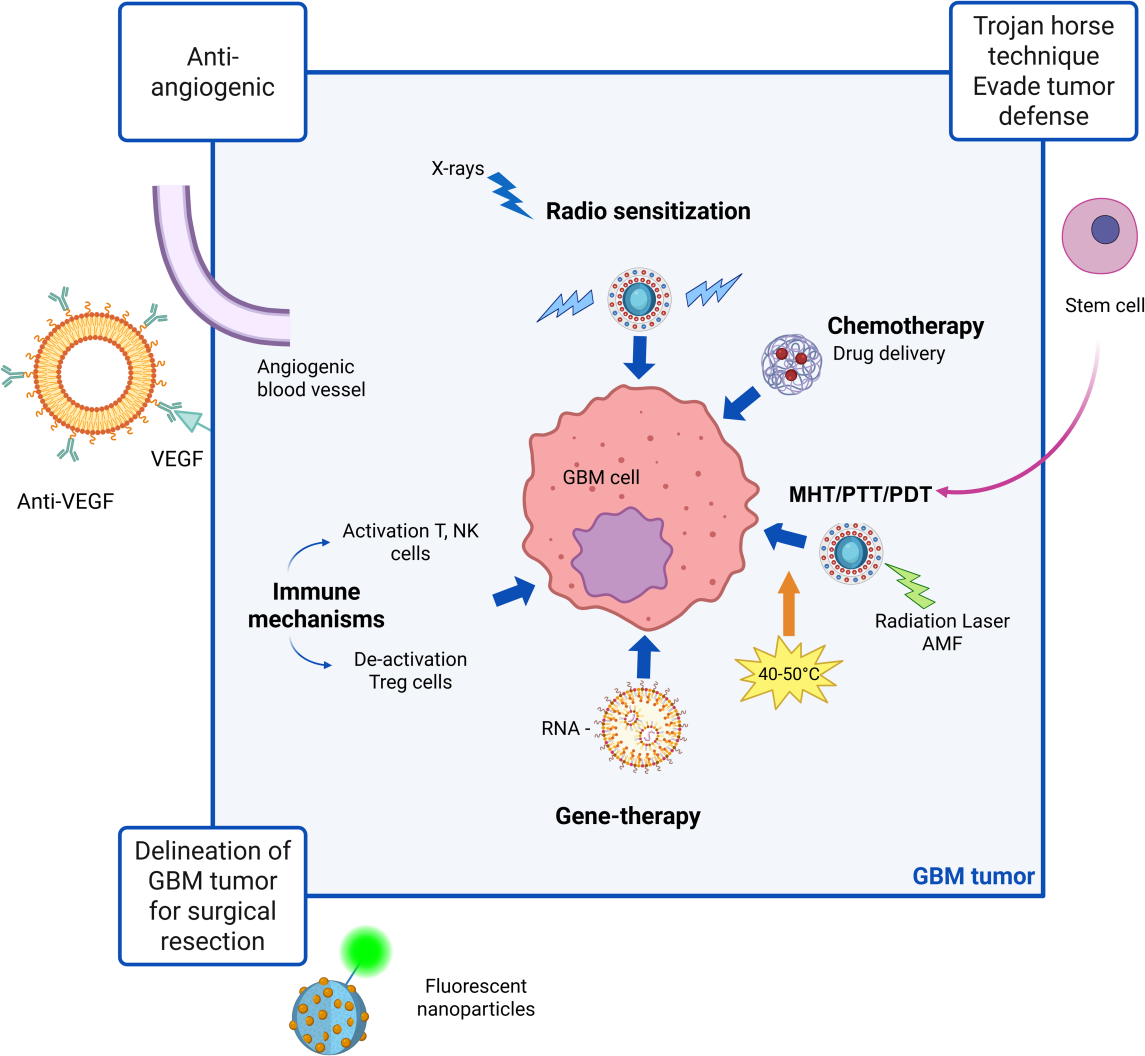
Schematic view of main action mechanisms of anti-GBM actively targeted and stimuli-responsive nano-drugs. Magnetic heating treatment (MHT), photothermal treatment (PTT) and photodynamic therapy (PDT) are specific strategies involving application of magnetic or radiation stimuli to the nanoparticles which may be synergistic in increasing drug delivery efficacy. Created in https://BioRender.com.

In brief, first-generation NPs nonspecifically targets tumor cells, second-generation focuses on active targeting through incorporation of ligands and specific antigens at NPs surfaces and third generation provides a multi-stage strategy best matching therapy and diagnostic (theranostic) purposes. Whereas first-generation nanotechnologies are clinical approved, second-generation platforms are currently being evaluated in clinical trials for combinatorial drug delivery strategies. Third generation instead have only recently emerged, primarily aimed at modulating immune responses and facilitating self-recognition mechanisms (16). First generation NPs mainly relies on Enhanced Permeability and Retention (EPR) effect due to leaky tumor vasculature and poor lymphatic drainage. These nano-systems are principally designed for improve the solubility and bioavailability of hydrophobic drugs and to protect encapsulated drugs from premature degradation, providing sustained release over time. Applications in GBM treatment concern the delivery of TMZ, DOX and PTX; main advantages are improved drug stability, enhanced solubility, prolonged drug action, simple design and manufacturing processes. Drawbacks of these nanotechnology are non-specific targeting and limited BBB penetration. In fact, although the BBB is disrupted at the tumor core, it remains largely intact in infiltrated brain regions, limiting systemic drug access to invasive GBM cells that drive recurrence after surgical resection (17). Second-generation NPs, retaining benefits of first-generation, add the ability to actively target glioblastoma cells. These NPs are designed to incorporate features as active targeting ligands and they rely on biocompatible surface modifications to improve specificity, reduce off-target effects, and enhance drug delivery to tumor cells. Finally, third-generation NPs are stimuli-responsive systems releasing payloads upon internal triggers (pH, redox, enzymes) or external stimuli (magnetic fields, light, ultrasound), combining therapeutic and diagnostic (theranostic) capabilities for real-time monitoring and treatment. They rely on techniques like magnetic guidance, receptor-mediated transport, ultrasound, as well as computed tomography (CT), Near InfraRed (NIR) and Magnetic Resonance (MR) imaging (18), photodynamic therapy (PDT) (19) and radiosensitization (20). The therapeutic protocol in PDT involves delivery of a photosensitizer (PS), followed by illumination of the target tissue with wavelength-specific light whereas radio-sensitization involves the use of X-rays. Ligands, antibodies, or peptides are used for receptor-specific targeting. As a resume, **Figure 1** presents a schematic view of the main action mechanisms of anti-GBM actively targeted and stimuli-responsive nano-drugs. The different ways of NPs-based intervention are depicted. Beyond strategies as antiangiogenic, immune mechanisms and gene-therapy, chemotherapy maintain a central role. Examples of NPs platforms along the three generations, as well as their applications and advantages in GBM treatment are resumed in **Table 2**.

**Table 2:** Generations of NPs for GBM: structures, delivered drugs and pros.

NP backbone	NPs structure	Drug	Pros	Ref
First generation				
Liposomes	Spherical vesicles - lipid bilayers encapsulating hydrophilic and hydrophobic drugs	TMZ	improve drug accumulation in tumor while reducing the drug exposure in blood and normal tissue	Zhan et al., 2019 (21)
	Cationic lipid nanoparticles containing covalently conjugated β-amphetamine in head-group	PTX	benefiting of BBB disrupting properties of lipophilic psychostimulants amphetamine	Saha et al., 2020 (22)
Polymeric	poly (lactic-co-glycolic acid) (PLGA) nanoparticles overcoated with poloxamer 188 (P188)	DOX	enable efficient DOX delivery inside glioma cells	Malinovskaya et al., 2017 (23)
	TerPolymer-Lipid-hybrid Nanoparticles (TPLN)	DOX	enhanced anticancer efficacy and GBM penetration and accumulation	Ahmed T et al., 2021 (24)
	Polybutylcyanocrylate (PBCA) NPs coated with polysorbate 80	CisPt	High retention capability and stability; efficacy in reducing side effects	Ebrahimi Shahmabadi H et al., 2014 (25)
	Cisplatin- Loaded Poly (Butylcyanoacrylate)	CisPt		Chiani M et al., 2019 (26)
	poly(lactideco-glycolide) NPs coated with poloxamer 188	DOX	enable brain delivery of this cytostatic that cannot penetrate across BBB in free form	Maksimenko O et al., 2019 (27)
	poly (lactic-co-glycolic acid) nanoparticles (PLGA NPs) coated with polyvinyl alcohol (PVA) and Poloxamer188 (P188)	MTX and PTX	promising for the treatment of glioblastoma compared to respective free drug formulations	Madani F et al., 2020; 2024 (28, 29)
	methoxy poly(ethylene glycol)-poly (lactic-co-glycolic acid) nanoparticles (mPEG-PLGA NPs)	PTX and etoposide (ETP)	enhanced antitumor efficacy, significant tumor regression, long-term survival	Maleki H et al., 2021 (30)
	Polyethylene glycol (PEG) modified and chitosan-coated PLGA NPs	PTX and R-flurbiprofen	mixed results on drug uptake and toxicity	Caban-Toktas S et al., 2020 (31)
	PLGA NPs	Carboplatin	greater tumour cytotoxicity, reduced neuronal toxicity and prolonged tissue half-life	Arshad A et al., 2015 (32)
	monomethoxy (polyethylene glycol) - poly (D, L-lactide-co-glycolide) (mPEG-PLGA).	TMZ & PTX co-loaded	mPEG help NPs escape from reticuloendothelial system phagocytose, prolong circulation, increase NPs accumulation at tumor through EPR effect	Xu Y et al.*, 20*16 (33)
Inorganic	magnetic γ-Fe_2_O_3_ NPs coated with P(HPMAH)-Dox conjugate	DOX	Significantly decreased the GaMG and GBM cell growth compared to free Dox; rapid division of tumor cells, enhanced cell permeation, increased cell death, reduced nonspecific toxicity	Plichta Z et al., 2020 (34)
	Silver NPs	CisPt	combination of AgNPs and CisPtP showed a favorable action via the stimulation of TRPM2	Akyuva Y et al., 2023 (35)
	mesoporous silica pegylated nanoparticles MSN-PEG NPs	PTX & Safranin-O	effective in slowing down the tumor’s regrowth *in vivo* increasing the survival of mice bearing U87 tumors; improving welfare	Erthal LCS et al., 2023 (36)
	iron oxide nanoparticles (IONPs) with attached carboxyl groups with high drug loading efficiencies	Cisplatin & siRNA	increased Fe^2+^ and Fe^3+^ levels; Fenton reaction between Fe^2+^, Fe^3+^ and intracellular H_2_O_2_ generated ROS species to initiate ferroptosis, co-released si-GPX4 inhibited GPX4 expression and synergistically improved therapeutic efficacy	Zhang Y et al., 2020 (37)
Natural bioactive molecules	Ursolic Acid NPs	PTX	inhibit proliferation of GBM cells and block cell cycle	Li Y et al., 2024 (38)
	pH-sensitive bio-mimetic NPs with acetal grated dextran inner core coated with GBM cancer cell membrane (CCM)	TMZ/CisPt	Natural cell membrane camouflaged NPs improve combinatorial treatment efficacy	Zou Y et al., 2022 (39)
Second generation				
Transferrin-Conjugated	TfR-targeted PLGA Nanoparticles Poly(lactic-co-glycolic acid) (PLGA) nanoparticles conjugated	TMZ	increase BBB penetration and tumor targeting	Mao J et al., 2019 (40)
	amphiphilic poly (γ-glutamic-acid-maleimide-co-L-lactide)-1,2-dipalmitoylsn-glycero-3-phosphoethanolaminecopolymer conjugated with targeting moiety transferrin	PTX	Tf-NPs enhanced the cytotoxicity of PTX in glioblastoma C6 cells	Wang L et al., 2021 (41)
	Transferrin-Modified Porous Silicon Nanoparticles	DOX	significantly enhanced cytotoxicity to GBM cells across an *in vitro* BBB monolayer compared with free Dox	Luo M et al., 2019 (42)
	PmAb-conjugated PLGA NPs. PmAb on surface functionalized using ethyl(dimethylaminopropyl)- carbodiimide (EDC)−N-hydroxysuccinimide (NHS)	TMZ	more pronounced anticancer effect in comparison with free TMZ and TMZ-PLGA-NPs.	Banstola A et al., 2020 (43)
RGD-modified	target integrin receptors αvβ3, highly expressed in angiogenic blood vessels	PTX	promote targeted delivery, reduce tumor growth improved accumulation	Wang L et al., 2021 (44)
	arginyl-glycyl-aspartic tripeptide (RGD) conjugated PLGA NPs	PTX	cancer-specific delivery; enhanced anticancer effects *in vivo*; intranasal treatment	Ullah I et al., 2020 (45)
	RGD-conjugated PLGA NPs	DOX	Cancer-specific delivery, inhibition of brain tumor growth; apoptosis in tumor region without affecting normal cells	Chung K et al., 2020 (46)
anti-ephrin receptor	anti-ephrin type-A receptor 3 (EphA3) modified Au-NPs	TMZ	active intranasal deliver enhanced cellular uptake; no tissue damage	Wang L et al., 2021 (47)
	anti-ephrin type-A receptor 3 (EphA3) modified PLGA-NPs	TMZ16e Temozolomide hexadecyl ester	reverse drug resistance *in vivo* enhanced median survival time	Wang S et al., 2022 (48)
inorganic	Iron Oxide nanoparticles (IONPs)	PTX	inhibits glioblastoma by enhancing autophagy-dependent ferroptosis pathway	Chen H et al., 2022 (49)
Functionalized natural bioactive molecules	CD44-Targeted Hyaluronic Acid Nanoparticles	DOX	selective cytotoxicity toward GBM cells expressing CD44	Zhang J et al., 2016 (50)
	Naringenin-loaded solid lipid NPs	PTX	significant improvement in drug absorption, higher cytotoxicity, superior uptake	Wang L et al., 2021 (44)
	Ascorbic acid conjugated	PTX	release pattern, increased concentration	Deshmukh V et al., 2025 (51)
Lactoferrin-conjugated	Pegylated Large pore silica nanoparticles	TMZ	efficient delivery	Janjua TI et al., 2023 (52)
Third generation				
Stimuli enhanced activation of inorganic NPs	biocompatible magnetic iron oxide nanoparticles (IONPs) stabilized with trimethoxysilylpropyl-ethylenediamine triacetic acid (EDT) EDT-IONPs	DOX	overcome both BBB and multidrug resistance; provides site-specific magnetic targeting	Norouzi M et al., 2020 (53)
	Fe_3_O_4_ and SiO_2_-coated with Fe_3_O_4_ MNPs for combined chemotherapy and photo-thermal heating	TMZ & ICG	chemo-photothermal therapy exhibited notable anti-cancer effects against U87 MG cells	Kwon YM et al., 2019 (54)
	multifunctional lipid-based magnetic nanovectors functionalized with peptide angiopep-2 responsive to alternating magnetic fields stimulation	TMZ	enable magnetic hyperthermia in synergy with chemotherapy	Beola L et al., 2023 (55)
	hybrid Gd^3+^ complexes grafted onto PEO micelle corona carboxylate functions at surface of micelle’s core able to cross link PT(II) complex.	CisPt	allow MRI monitoring distribution in the parenchima and its therapeutic benefit	Lajous H et al., 2018 (56)
	MIL-Modified Fe3O4 NPs with increased loading capability due to highly porous metal-organic framework	TMZ	low toxicity and increased adsorption capacity than bare MNPs. Enhanced Uptake and Efficiency in Glioblastoma Treatment	Pulvirenti L et al., 2020 (57)
	Lanthanum Oxide (La_2_O_3_) NPs	TMZ	lanthanum cytotoxic to cancers. Can reach the brain after venous injection, penetrate into GBM cells via endocytosis, dissociate to be cytotoxic, enhance therapeutic effects of RT and TMZ	Lu VM et al., 2020 (20)
	Pt-Fe Nanostructured Coordination Polymers (NCPs)	Pt (IV)	Reduce cytotoxic side effects due to precisely controlled release. Enable MRI to measure tumor location and evolution	Mao X et al., 2022 (58)
	Gold NPs	DOX, PTX, TMZ CisPt	easy synthesis, biocompatibility, surface functionalization, ability to cross BBB. Applied in imaging, diagnosis, photothermal therapy, radiotherapy	Neshastehriz A et al., 2018 (59) Jing Z et al., 2021 (60) He C et al., 2021 (61) Depciuch J. et al., 2020 (62)
	SPIO (Super paramagnetic Ion Oxide) NPs loaded in RGD-grafted PLGA	PTX	MRI imaging, magnetic targeting strategy allowed a better chemotherapeutic effect	Ganipineni LP et al.*, 20*19 (63)
	Cu_2-x_Se nanoparticles	DOX	Combined PDT and chemotherapy	Zhang H et al., 2019 (19)
	Cit/CuS@ Fe_3_O_4_-based and enzyme-responsive magnetic nanoparticles	DOX	Combined chemotherapy, photothermal and photodynamic therapy; higher antitumor efficacy than individual therapies *in vitro* and *in vivo*	Zhu X et al., 2017 (64)

Polyethylene glycol (PEG) or poly(lactide-co-glycolide) (PLGA) are a commonly used coating agent in nanomedicine (23, 27, 28, 30–33). These polymers FDA-approved for human medical applications show biodegradability, biocompatibility and non-toxicity properties. NPs derived from PLGA can be prepared by well-assessed methodologies, as nano-precipitation methods, and they may be coated or grafted with a variety of moieties. Polyethylene glycol (PEG) conjugation is particularly advantageous as it mitigates nanoparticle hydrophobicity by imparting a hydrophilic steric barrier on the surface (30, 33). PEG-PLGA nanoparticles have demonstrated significant efficacy in the co-delivery of two or more therapeutic agents (30). Metal-based nanoparticles are promising cancer therapies, and recent studies have focused on their applications yielding the following types: (i) gold (59); (ii) silver (35); (iii) iron oxide (37, 49, 54); (iv) Magnetic-nanoparticles (20, 53, 63) to profit of MHT effect; (v) mesoporous silica nanoparticles (MSN) (36), where porous surface functionalization enables strategic pore closure to regulate drug release and achieve targeted delivery to specific sites (vi) nanocarriers acting as photosensitizers as Cu_2-x_Se (19) to combine PDT with chemotherapy; (vii) NPs cytotoxic to cancer sensitizing glioblastoma cells to radiation therapy and temozolomide (20).

### 2.2 Morphological issues in nanoparticle systems for GBM

**Figure 2:**
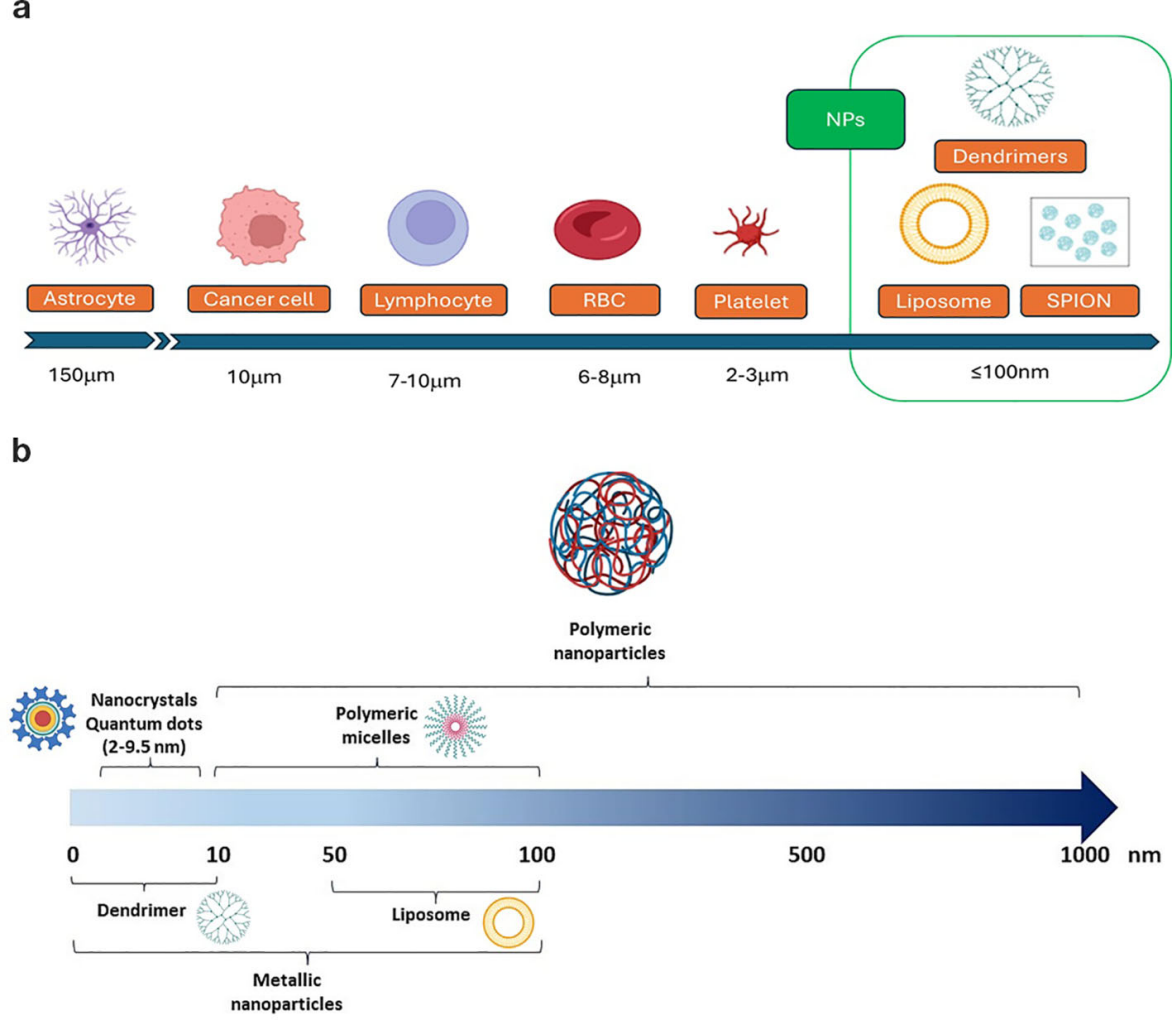
Typical size of **(a)** body cells and of **(b)** some of the nanoparticle systems involved in GBM therapy.

Size and surface characteristics as size, shape, porosity, charge are critical factors influencing the NPs drug delivery effectiveness. To investigate these parameters, nanoparticles are usually characterized by standard methods, using transmission and scanning electron microscopy (TEM and SEM) before and after surface functionalization, along with powder X-ray diffraction (PXRD) and infrared (IR) spectra. For SEM analysis, non-metallic NPs are coated with a gold/palladium thin layer under vacuum. Dynamic light scattering (DLS) and Nanoparticle Tracking Analysis (NTA) are commonly used to determine the mean particle size and size distribution of nanoparticles, and the zeta potential of suspended particles using a zeta potential analyzer. The technique involves using electrophoretic light scattering, also known as laser Doppler electrophoresis. Surface area and pore size distribution are analyzed by nitrogen adsorption–desorption porosimetry techniques. A sketch of typical NPs relative size versus other cells of the body are reported in **Figure 2**.

The small size of nanoparticles (NPs) is an advantage for crossing the blood-brain barrier (BBB). Studies have shown that the BBB permeability through gaps increases as NP size decreases, with minimal permeability for particles larger than 200 nm, nonetheless, renal filtration rapidly clears NPs < 5 nm, so typical sizes are within 10 to 100 nm (65). Diameters ranging between 10 nm and 40 nm are ideal for prolonged circulation in the bloodstream, increasing their efficiency in targeting the tumor microenvironment and crossing the blood-brain barrier. Smaller sizes ensure better diffusion and deeper penetration into the glioblastoma tissue. Nanoparticles may be characterized by a tiny core surrounded by structures of higher size. As an example, in (22) the diameter of the PLGA NPs without any coating, about 50 nm, increases to about 84 nm with PVA/P188 coating and a further increase is observed after drug co-loading (about 200 nm). A smaller core size is a general characteristic of iron oxide nanoparticles used in biomedical applications, in the range 5 nm to 60 nm, and a hydrodynamic size diameter up to 100 nm. Due to their small size, these cores cannot form stable magnetic domains, leading to the phenomenon of superparamagnetism. Single-domain nanoparticles exhibit uniform magnetization, and when exposed to a magnetic field, their magnetic moments align with the field direction. Superparamagnetic nanoparticles therefore do not exhibit remanence or coercivity, and their magnetic moments disappear when the magnetic field is switched off. The behavior of such super-paramagnetic ions (SPIONS) is like paramagnetic substances, but with much larger magnitude. This property allows to stabilize iron oxide magnetic nanoparticles in the target area using an external magnetic field, enhancing targeted delivery while minimizing systemic toxicity (16). For instance, in (37), a system based on porous IONPs functionalized with carboxyl groups was developed to naturally bind iron, initiating the Fenton reaction, while si-GPX4 loading amplified ferroptosis-mediated antitumor activity.

Directly correlated with the size, polydispersity index (PDI), defined as the square of the nanoparticle population standard deviation over the nanoparticle mean diameter, characterizes the spread width of the nanoparticle size distribution profile. For a most uniform population, PD should be as close as possible to 0, while FDA’s guidelines suggest that PDI should remain below 0.3. As an example, excellent uniformity is reported for co-loaded Paclitaxel/methotrexate PLGA nanoparticles, with PDI 0.04 to 0.12 (28) while a significantly high PDI (0.4) was reported for mesoporous silica nanoparticles in (36).

Zeta potential is the electrical potential difference between a nanoparticle’s surface and the surrounding liquid. It is closely related to surface charge and significantly impacts drug delivery. Nanoparticle’s net surface charge is surrounded in an ionic solution by a tightly bound layer of oppositely charged ions, as well as a loosely associated electrical double layer. As the nanoparticle moves, some ions within the diffuse layer travel with it, while others remain behind. A high zeta potential, indicating stronger electrostatic repulsion, can enhance nanoparticle stability by preventing aggregation. In contrast, neutral or weakly charged nanoparticles typically exhibit reduced stability.

The surface charge is in general modified by surfactant or active molecule adsorption, PEGylation, by coating the surface and conjugating targeting ligands. For instance, mPEG-PLGA NPs in (30) characterized by a -22mV zeta-potential, are the range of excellent colloidal stability. Moreover, cationic molecules can traverse the barrier *via* adsorption-mediated transcytosis, enabled by electrostatic attraction to the negatively charged endothelial cell plasma membrane. With this aim, cationic lipid nanoparticles have been considered in (22). Zeta-potential may change after drug loading. As a good example, EDT-coated IONPs in (53) uses EDT as a biocompatible coating providing many negatively charged sites on the surface of the NP that can interact with the positively charged DOX molecules. In fact, DOX-EDT-IONPs resulted in a zeta potential of 0.0 mV compared to – 20 mV for EDT-IONPs. This shift in surface charge following drug loading is due to the electrostatic interactions between the amine groups of DOX and carboxylic acid groups of EDT coating.

In general, nanoparticles with a high zeta potential (ζ ≥ ± 10 mV), due to their strong electrostatic repulsion, are more stable and less prone to aggregation (66). Other important parameters are the encapsulation efficiency, EE%, namely the ability to encapsulate the drug within the NP system, and drug loading, amount of total drug loaded over the total nanporaticle weight: $EE\%=\;\frac{encapsulated\;drug\;}{total\;drug\;added}$ , $DL\%=\;\frac{drug\;loaded\;weight\;}{total\;nanoparticle\;weight}$ , parameters estimated using High-Performance Liquid Chromatography (HPLC), see e.g. (36). Typical physicochemical ranges for nanocarriers in glioblastoma drug delivery are reported in **Table 3**.

**Table 3:** typical physicochemical ranges for nanocarriers in glioblastoma drug delivery.

Parameter	Technique	Range	Reference
Size	SEM, TEM, AFM	10–100 nm ≈ 5 nm	Hersh (65) Norouzi (53)
Hydrodinamic diameter	DLS, NTA	≈ 200 nm ≈ 50 nm	Saha 2020 (22) Norouzi (53)
Polydispersity index	DLS	0.04 to 0.12 0.40 0.14	Madani 2020 (28) Erthal 2023 (36) Norouzi (53)
*ζ*-potential	*ζ* -potential analyzer	≥ ± 10 mV -8 ÷ -24 mV -22 mV -27mV	Oztürk (2024) (66) Madani 2020 (28) Maleki 2021 (30) Norouzi (53)
Encapsulation efficiency	HPLC	70-90% 92% 20% 80% 67%	Madani 2020 (28) Maleki 2021 (30) Zhang (2020) (37) Malinovskaia 2020 Erthal 2023 (36)
Drug Loading	HPLC	8-12% 18%	Madani 2020 (28) Erthal 2023 (36)

## 3 Nanoparticle-loaded with cytotoxic chemotherapeutic drugs

Despite TMZ being the sole chemotherapeutic agent currently used in glioblastoma treatment (as first line) there are a lot of other promising therapeutic cargoes for nanoparticles. Several classic antitumor agents (such as TMZ, PTX, CisPt, DOX) have been delivered to GBM using many types of nanosystem. First-generation nanocarriers use passive targeting based on the EPR effect, but recent research focuses on adding active targeting to better direct drugs to tumors. Solid lipid nanoparticles, polymeric nanoparticles, micelles, gold nanoparticles, super-paramagnetic iron oxide nanoparticles (IONs) and mesoporous silica nanoparticles are the most widely used at this scope. Herein a short overview of the recent findings on improvement in delivery classical chemotherapeutic drugs and their shortcomings and progress.

### 3.1 Temozolomide

Temozolomide is an oral alkylating prodrug of 3-methyl-(triazen-1-yl) imidazole-4-carboxamide (67). In 2005, a landmark phase III trial described its efficacy and, since then, temozolomide has become the first-line chemotherapeutic agent for treating malignant glioma (68, 69). Nevertheless, TMZ faces pharmacokinetic challenges, including a short plasma half-life (~2 hours), susceptibility to P-glycoprotein (P-gp) efflux, and limited penetration across the blood-brain barrier (~20%) (70). Organic and inorganic nanoparticles and passive vs active targeting has been used to improve TMZ pharmacokinetics and pharmacodynamics properties. Although TMZ can partially cross the blood–brain barrier to exert its therapeutic effect, the high systemic doses required often lead to severe side effects (70). At first, temozolomide-loaded PLGA nanoparticles have failed to improve cytotoxicity in U87MG cultures (TMZ alone vs TMZ-PLGA NPs) (71). Then, PLGA-nanoparticles active targeting by both overexpression of Epidermal Growth Factor Receptor (EGFR) and transferrin-conjugated (TfR) enhanced cellular uptake of TMZ alone and in combination with Bortezomib (BTZ) (43, 72). Notably, unmodified nanoparticles were more effective at inhibiting tumor cell growth than transferrin-conjugated (TfR) nanoparticles. This is likely due to their faster drug release rate, which promotes increased intracellular drug accumulation. Next, a powerful synergistic approach, based on intertumoral chemo-hyperthermia induced a potent TMZ anti-glioma effect. In this study, lipid-based magnetic nano-vectors functionalized with angiopep-2 peptide (Ang-TMZ-LMNVs) enhanced the specificity in an orthotopic human GBM mouse model. They accumulate in tumors without affecting normal tissue, inhibiting tumor growth and extending life (median survival time doubled) when combined with an alternating magnetic field (55). Inorganic nanoparticles were efficiently delivered through ultrasmall, large-pore silica nanoparticles (USLP), which are PEGylated silica nanoparticles with lactoferrin as a tumor-targeting moiety. *In vitro* assays demonstrated increased cytotoxicity against human and mouse cell lines, and a 3D spheroidal model revealed decreased TMZ efflux across the blood-brain barrier (BBB) measured by trans-endothelial electrical resistance. *In vivo* studies confirmed that the USLP delivery system accumulates in the brain (52). Metallic iron oxide IONPs Fe_3_O_4_ TMZ-loaded obtained an excellent photothermal effect by synergistic combination of a chemo-phototherapy approach, increasing the anticancer effects in human glioblastoma cells by enhanced ROS generation (54). Similarly, magnetic Fe_3_O_4_ hybrid nano-system enhanced uptake and efficiency of TMZ treatment by a co-precipitation method (57). A proof-of-concept study using lanthanum oxide (La_2_O_3_)-based nanoparticles was performed exclusively on patient-derived glioblastoma cell lines, successfully overcoming the unavailability of experimental data for non-immortalized cells. Lanthanum oxide (La_2_O_3_) nanoparticles offer therapeutic benefits by virtue of lanthanum’s unique chemical properties. This study presents *in vitro* evidence that cytotoxic La_2_O_3_ nanoparticles sensitize glioblastoma cells to both radiation therapy and temozolomide (20). Inorganic gold nanoparticles have been used for the active TMZ delivery in glioblastoma by intranasal delivery. The direct neural connections (olfactory and trigeminal pathways) permit to bypass the blood-brain barrier, enabling efficient delivery of nanoparticles directly to the brain. In central nervous system diseases, including brain cancer, it can improve drug bioavailability, reduce systemic side effects, and allow non-invasive administration. *In vitro* studies showed that anti-EphA3-TMZ@GNPs significantly enhanced cellular uptake and toxicity toward both human and rat cell lines. To evaluate intranasal administration, an orthotopic glioma-bearing rat model was employed to perform a comprehensive *in vivo* safety assessment (47). More, intranasal administration was used in another study with TMZ-hexadecyl ester (TMZ16e). Anti-ephrin type-A receptor 3 (EphA3) modified TMZ16e loaded nanoparticles (NPs) were found to improve brain targeting efficiency and anti-glioma activity, as well as reverse TMZ resistance (48).

### 3.2 Paclitaxel

Paclitaxel (PTX) is a widely recognized and potent chemotherapeutic agent used to treat peripheral solid tumors (73). PTX suffers from poor BBB penetration and early trials failed. Recently, optical blood-brain-tumor barrier modulation expands treatment options with vascular-targeted gold nanoparticles, significantly enhancing the PTX delivery in GEMM (genetically engineered mouse model) (74). Many PLGA-based NPs has been designed to overcome the limitations of the systemic delivery of PTX. Combination of PTX and R-flurbiprofen loaded PLGA nanoparticles suppresses glioblastoma growth on systemic administration, by *in vitro* and *in vivo* assays. This work is the only pre-clinical study that use a nonsteroidal anti-inflammatory drug, R-Flurbiprofen, in association with PTX. The experiments on rat model RG2 and in brain-tumorized rats obtained mixed results on drug uptake and toxicity, but remarkably this combination reduced inflammation in the peri-tumoral area (31). Organic PLGA nanoparticles were co-loaded with methotrexate or etoposide and PTX in two similar studies. *In vivo* orthotopic glioblastoma-bearing rats, treatment with PTX/ETP co-loaded mPEG-PLGA NPs resulted in enhanced antitumor efficacy with significant tumor regression and a long-term survival in 40% of the animals. Noteworthy, hemocompatibility assays confirmed the blood safety of this approach (30). Further, surface-modified (with poloxamer 188) nanoparticles PLGA co-loaded with paclitaxel/methotrexate enhanced survival rates, showed better neurological outcomes, and favorable histopathological profiles of major organs (28, 29). Recent studies have shown that both ferroptosis and autophagy are key mechanisms in cancer therapy. An example, iron oxide nanoparticles (IONP@PTX) PTX-loaded inhibited glioblastoma by enhanced autophagy-dependent ferroptosis. IONP@PTX inhibited cell viability, migration and invasion *in vitro* and decreased the tumor volume of GBM xenografts *in vivo* (49). Efflux transporters (i.e. ABC superfamily) include also P-glycoprotein (P-gp) as key contributors to chemotherapy failure through multidrug resistance mechanisms. P-gp/ABCB1 is found to be highly expressed in cerebral vascular endothelial cells and brain tumors, and, notably, PTX is mainly eliminated through this efflux pump. An interesting study describes Ursolic Acid Nanoparticles (UA NPs) as effective inhibitors of P-gp transporters that enhance the delivery and efficacy of PTX in glioblastoma, showing an excellent biocompatibility *in vivo* (38). Another type of organic-based NPs (cationic lipid nanoparticle), amphetamine-functionalized PTX-loaded efficiently crossed the BBB and combined with PD-L1 siRNA synergistically improved survival in glioblastoma-bearing mice (22). Similarly, peptide-modified [arginyl-glycyl-aspartic tripeptide (RGD)] PTX-loaded NPs showed cancer-specific intranasal delivery, enhanced *in vitro* anticancer effects and reduced tumor growth *in vivo* (45).

Leveraging the active targeting of αvβ3 integrin, overexpressed in both neoangiogenic vasculature and glioblastoma cells, magnetic targeting PLGA-based NPs improved accumulation in the tumors. PTX-loaded RGD-multifunctional nanoparticles showed *in vivo* safety and anti-tumor efficacy on U87MG cultures (63). A novel local chemotherapy formulation, GlioGel—comprising free temozolomide (TMZ) and paclitaxel (PTX)-loaded PEGylated mesoporous silica nanoparticles (MSNs)—was developed and tested both *in vitro* and in a preclinical GBM mouse model following tumor resection. *In vivo*, MSNs effectively delayed tumor regrowth, prolonged survival in U87 tumor-bearing mice, and improved overall welfare (measured by the pattern of body weight change over time) (36). Combined delivery of paclitaxel (PTX) and naringenin, a bioactive phytocompound with anticancer properties, were obtained by solid lipid NPs. RGD-conjugated formulations (cRGD; Gly-Asp-Arg) significantly improved the release rate and drug absorption performance, as shown by *in vitro* and *in vivo* detailed pharmacokinetic studies (44). Transferrin-conjugated improved tumor targeting of PTX-loaded NPs and showed synergistic effects through transferrin-mediated endocytosis and high biocompatibility in a rat glioma model (41). More recently, intranasal delivery of surface-modified (AA Ascorbic Acid) PTX-loaded polymeric nanoparticles (AA-PTX-PNPs) increased PTX concentration. This study reports an accurate characterization by *in vitro* and *in vivo* pharmacokinetic assays showing a biphasic release pattern and a minimal alteration by histopathological results (51).

### 3.3 Cisplatin

Cisplatin (CisPt) is one of the most potent antitumor agents known, displaying clinical activity against a wide variety of solid tumors outside the central nervous system. Systemically delivered CisPt penetrates poorly into normal brain tissue due to the BBB. Although cisplatin is cytotoxic on human glioblastoma cells *in vitro*, the response in clinical treatment is weak and has not improved the overall survival of patients with brain tumors. Moreover, its efficacy is often limited by the development of resistance (75).

Hence, cisplatin-loaded nanoparticles are poorly represented in the current literature. Some examples of cisplatin carrying nanoparticles are listed below. Iron oxide nanoparticles (IONPs) were used for codelivery of CisPt and GPx in immortalized cultures and patient-derived glioblastoma. They enhanced therapeutic efficacy synergistically *via* a ferroptosis-related mechanism (37). Another study demonstrated that inorganic hybrid gadolinium nanoparticles exhibited up to a 50-fold increase in accumulation within human glioblastoma cells, along with a 32-fold enhancement in Pt-DNA adduct formation compared to free cisplatin. Additionally, non-invasive MRI tracking of nanoparticle biodistribution confirmed the potential of this innovative bimodal platform for applications in nuclear medicine (56). A similar approach has been developed by Mao et al. for intranasal administration of Catechol-Based Pt (IV) coordination polymer. MRI monitoring demonstrated enhanced tumor accumulation, resulting in complete remission and extended survival outcomes as measured by Kaplan–Meier curves (58). Some efficacies of CisPt-loaded poly-Butyl cyanoacrylate NPs were reported both *in vitro* and *in vivo* rat model of glioblastoma (25, 26). More recently, brain co-delivery of TMZ and CisPt has been proposed as a combinatorial glioblastoma chemotherapy. These biomimetic nanoparticles MNPs@TMZ+CisPt exhibited a potent anti-GBM activity in a mice orthotopic model (39). Carboplatin, a less toxic analogue of cisplatin, has been used in a study using convection-enhanced delivery with PLGA nanoparticles (32). Alginate nanogel CisPt co-loaded with gold nanoparticles and silver nanoparticles has been used to potentiate the oxidant actions of CisPt *via* the stimulation of TRPM2 channel in glioblastoma cells (35, 59).

### 3.4 Doxorubicin

Doxorubicin (DOX) is an anthracycline antitumor drug discovered in 1969. DOX was among the first chemotherapies encapsulated in a cell membrane-cloaked polymeric nanoparticle (76). One of most used chemotherapeutic agents for treating both solid and hematologic malignancies. Covalent linkage of DOX to three different types of NPs-metallic, silica/organo-silica and polymeric has been shown to overcome its cardiotoxicity (77).

Intranasal delivery of DOX-loaded PLGA NPs arrests growth in rat model of glioblastoma. In this study, PLGA nanoparticles NPs were modified with the RGD arginyl-glycyl-aspartic tripeptide (RGD) ligand to enable active targeting of αvβ3 integrin. Moreover, its intranasal administration enhanced apoptosis in the tumor area, without harming normal brain tissue (46). Two examples of organic DOX-loaded PLGA confirmed the nanoparticles’ anti-tumor efficacy: (i) a pilot-scale manufacturing process yielded strong anti-tumor efficacy in *in vivo* orthotopic model, with negligible blood toxicity at therapeutic concentrations (27) and (ii) a detailed confocal characterization of intracellular trafficking of DOX-loaded PLGA NPs in human U87MG describing NPs internalization by clathrin-mediated endocytosis (23).

Many examples of second-generation nanostructure reports DOX−loaded inorganic metal nanoparticles for glioblastoma therapy. Nourouzi et al. report a combinational approach for enhanced delivery of iron oxide nanoparticles (IONPs). A cadherin binding peptide with trimethoxy silyl propyl-ethylenediamine triacetic acid (EDT) and an external magnetic field enhanced the NPs penetration and increased therapeutic response and apoptosis in human U251 cells (53). Plichta et al. moved one step further using five patient-derived primary glioblastoma cultures for cellular assays. Poly[N-(2-hydroxypropyl) methacrylamide]-modified magnetic γ-Fe_2_O_3_ nanoparticles Dox-conjugated were proposed as a blood plasma substitute instead of PEG for glioblastoma treatment (34). A detailed study reports terpolymer-lipid hybrid nanoparticle (TPLN) developed for DOX delivery to GBM, demonstrating enhanced *in vitro* and *in vivo* efficacy measured by cellular uptake, cytotoxicity, 3D spheroid penetration, and biodistribution in a murine orthotopic GBM model (24). A study exclusively performed on brain microvascular cells (instead of an orthotopic glioma model), reports active targeting by transferrin-modified porous silicon nanoparticles Tf@pSiNPs in *in vitro* monoculture U87MG and coculture BBB model describing clathrin- and caveolae-endocytic pathways (42). Again, CD44-targeted and redox-responsive drug delivery system was based on mesoporous silica nanoparticles (MSNs) exhibited higher cellular uptake efficacy *via* CD44-mediated endocytosis and higher cytotoxicity (50).

## 4 Combining PDT/RT and chemotherapeutic NPs in GBM treatment

Nanoparticles for photodynamic therapy (PDT) are approved by the US Food and Drug Administration (FDA) for many cancers (78). 5-ALA is the only fluorescence-guided glioma surgery agent approved by the FDA (79). Integration of Photodynamic therapy (PDT) and Radioterapy (RT) to chemotherapeutic NPs represents a novel strategy to enhance GBM treatment outcomes, to mitigate toxicities associated with individual agents and substantially enhance overall therapeutic efficacy (80). Photodynamic therapy (PDT) involves the administration of a photosensitizer (PS), either topically or systemically, followed by irradiation of the target tissue with a light source matched to the absorption wavelength of the PS. Photosensitizers that absorb in the visible or near-infrared spectrum are preferred due to their lower phototoxicity compared to ultraviolet light. Photodynamic therapy (PDT) may be utilized as a monotherapy or integrated with conventional treatment modalities, administered either prior to or following their application.

Several NP systems may serve a dual role as radio- or photosensitizers and drug delivery carriers. Polymersomes (or polymer vescicles) functionalized with angiopeptide-2 (Ang-2), loaded with gold nanoparticles (AuNPs), and conjugated with doxorubicin (DOX) (Au-DOX@PO-ANG) have demonstrated enhanced permeability across the blood–brain barrier (BBB) and selective accumulation in malignant brain tissue (61). Targeted delivery of this therapeutic platform to tumor sites significantly enhanced radiosensitization, resulting in a 40% decrease in cell viability post-radiotherapy, indicating a substantial cytotoxic effect. Near-infrared (NIR) imaging analysis revealed that rats receiving combined treatment with Au-DOX@PO-ANG and radiotherapy exhibited significantly reduced tumor growth and prolonged survival compared to controls. Additionally, the delivery system demonstrated high stability and no observable toxicity in major organs.

Besides classical drugs, AuNPs may also deliver other compounds that display antitumor effects. Gallic acid (GA) has been investigated as a potential anti-cancer agent in glioblastoma tumors (60). GA-AuNPs reduced U251 GBM cell viability by up to 31.25% particularly by day 3, and increased apoptosis. Treated cells showed S and G2/M phase arrest, with 150–200 µg/mL GA-AuNPs enhancing radiosensitivity across 2–12 Gy doses improving the efficiency of radiotherapy. Finally, gold nanopeanuts (AuNPes), owing to their unique shape and high surface area, exhibit enhanced drug-loading capacity (e.g., for cisplatin), making them promising candidates for combined chemo- and radiotherapy applications (62).

Some studies have recently investigated the efficacy of combining chemotherapy and PDT for glioblastoma treatment. Zhang and colleagues developed a Cu_2-x_Se-based nanoplatform for treating malignant glioblastoma through a combination of near-infrared photodynamic therapy and chemotherapy using doxorubicin as the therapeutic agent (19). Cu_2-x_Se nanoparticles exhibited strong infrared absorption at approximately 1064 nm, enabling deep tissue penetration. Additionally, they effectively catalyzed the degradation of H_2_O_2_ and intratumoral oxygen, generating substantial levels of reactive oxygen species (ROS) (81). A long-term issue for the limitation of PDT is represented by hypoxia. A promising tumor microenvironment-triggered oxygen nanogenerator for self-enhanced PDT primed antitumor immunotherapy has been designed in (82), using indocyanine green (ICG) PS and gold nanoshells in photothermal therapy (PTT) to promote the catalysis of hydrogen peroxide and self-enhance PDT. The relief of tumor hypoxia broke the chemoresistance and promoted the polarization of tumor-associated macrophages from M2 to M1 type, increasing the efficacy of chemotherapy and immunotherapy. In (83) MnO_2_-Ce6 nanoparticles have been applied for an effective combination of photodynamic and chemotherapy. They are designed to react with H_2_O_2_ in tumor microenvironment so to produce oxygen and thus overcome hypoxia-associated photodynamic resistance. Meanwhile, gradual decomposition into individual therapeutic albumin complexes improve intratumoral diffusion. As a result, effective *in-vivo* antitumor therapeutic outcome is obtained by a single treatment at a rather low dose. More recently, surface functionalized graphene quantum dots (GQDs) have been shown the capability to cross the blood–brain barrier and exert synergistic photodynamic and photothermal effects in combination with chemotherapeutic doxorubicin and temozolomide (84, 85). In particular, the capability of GQDs to absorb and convert near-infrared light into heat in PhotoThermal Therapy (PTT) enhanced membrane permeability, increasing the release of reactive oxygen species and ultimately the efficacy of antitumor drugs at subtherapeutic doses against glioblastoma.

Many examples exist that report nanoparticles as both nanocarriers and photosensitizers such as citric acid/CuS@Fe_3_O_4_ (64), zinc (II)phthalocyanine (86), cyclometallatediridium (III) (87), silver nanoparticles (88) and NaYF_4_: Yb/Tm (89). All nano-platforms demonstrated good *in vitro* and *in vivo* therapeutic efficacy. Nonetheless, they have not been applied to GBM.

Nanoparticle-enhanced radiotherapy represents an emerging frontier in the treatment of brain tumors (90). In general, NPs enhance the efficacy of RT by boosting the production of ROS, increasing oxidative stress and binding to DNA in terms of chemical interactions. High atomic number (Z) metal nanoparticles can enhance radiotherapy efficacy by targeting specific biological pathways. Upon irradiation, these nanoparticles emit secondary X-rays, photoelectrons, and Auger electrons, thereby amplifying the local radiation dose delivered to tumor tissues. Conversely, elevated biological responses in tumor tissues—such as oxidative stress and DNA damage—can potentiate the therapeutic effects of radiotherapy.

Lanthanum-based nanoparticles (La_2_O_3_ NPs) offer therapeutic advantages in glioblastoma treatment (20) due to their preferential accumulation in tumor cells over astrocytes and their ability to cross the BBB. When combined with radiotherapy or temozolomide, La_2_O_3_ NPs enhance apoptosis, DNA double-strand breaks, and autophagy by molecular mechanisms involving ROS/γ-H2AX signaling and Bcl-2 expression. (See **Table 4** application of nanocarriers in PDT/PTT/RT).

**Table 4:** Synergistic application of nanocarriers in PDT/PTT/RT and chemotherapy in cancer.

NP system	Drug	Synergy	GBM	Ref
MnO_2_ Ce6	Cis-Pt(IV)SA	PDT	Yes	(83)
GQDs	Dox, TMZ	PDT	Yex	(84)
GQDs	Dox, TMZ	PTT	Yes	(85)
Cu_2-x_Se	dox	PDT	Yes	(19)
Citric acid/CuS@Fe_3_O_4_	Dox-cit	PDT	No	(64)
zinc (II)phthalocyanine	Coumarin derivative	PDT	No	(86)
cyclometallatediridium(III)	camptothecin CPT	PDT	No	(87)
Ag NPs	DOX	PDT	No	(88)
NaYF_4_: Yb/Tm	DOX	PDT	Yes	(89)
Polymeric NPS/ rose bangal	DOX	PDT	No	(91)
Au NPs and ICT	Paclitaxel	PDT	No	(82)
La_2_O_3_ NPs	TMZ	radiotherapy	Yes	(20)
AuNPes	cisplatin	radiotherapy	Yes	(62)
AuNPs	GA	radiotherapy	Yes	(60)
thermosensitive liposomal (TSL)- IR820	Paclitaxel	PTT, PDT	No	(92)

## 5 Current clinical trials of drug-loaded nanoparticles in glioblastoma

Although extensive research explores nanoparticles as potential brain cancer therapies, only a few have gained approval from the FDA and EMA (93). This stems from an incomplete understanding of glioblastoma biology and a gap between preclinical drug development and clinical evaluation (94). Preclinical testing based on *in vitro* IC50 evaluation of chemotherapeutic drugs on glioblastoma cultures rely on sketchy and mixed results (*unpublished observations*). Many early clinical trials of chemotherapies and molecularly targeted treatments in patients with primary and metastatic brain tumors failed to produce patient benefits (1–3). Liposomal encapsulation technology showed limited physico-chemical stability due to fragile phospholipid membranes and their peroxidation and clinical trial using pegylated liposomal doxorubicin (Caelyx™, PEG-Dox) failed to produce a significant improvement in patient’s (95). In the era of the cancer nanomedicine, many formulations, examples such as Abraxane, NanoTherm, and Combidex—comprising both organic and inorganic nanoparticles—have received clinical approval or are currently in clinical trials for solid tumors, but very few for glioblastoma (96, **Table 5**).

**Table 5:** Summary table of major phase I-III ongoing clinical trials with drug-loaded nanoparticles in Glioblastoma.

Title of the study	Clinical phase	Study results	NCT number
Application of Nanoparticles for Cyclic Hyperthermia in Adjuvant Therapy of glioblastoma Multiforme (ANCHIALE) (NanoTherm)	Recruiting (Estimated Study Completion Date 2027-01)	No Results available	NCT06271421
AGuIX Nanoparticles with Radiotherapy Plus Concomitant Temozolomide TMZ in the Treatment of Newly Diagnosed Glioblastoma (NANO-GBM)	Active, not recruiting (Estimated Study Completion Date 2027-03)	No Results available	NCT04881032 (97)
INtraoperative photoDYnamic Therapy of GliOblastoma (INDYGO)	Recruitment Status: Completed	No Results available	NCT03048240 (Vermandel M et al., 2021) (98, 99)

Here we report only those based on classical delivery of chemotherapeutic drugs, not molecularly targeted (i.e. EGFR or other not-validated target gene). A phase I/II clinical trial (NANO-GBM) is currently ongoing to evaluate AGuIX nanoparticles combined with radiotherapy and concomitant TMZ for newly diagnosed glioblastoma, with the primary objective to establish the recommended dose of AGuIX combined with radiotherapy and TMZ during concomitant chemoradiotherapy, and to assess the efficacy of this combination by evaluating the 6-month progression-free survival (PFS) rate (phase II) (NCT04881032; 97, 100). The only EMA-approved brain cancer drug therapies based on nanotechnology is NanoTherm (MagForce). An iron oxide aminosilane-coated nanoparticles, in form of nanocrystal which has been registered in Europe (EMA) as a method of treating glioblastoma multiforme recurrence. This is a magnetic hyperthermia device (NanoTherm^®^ Therapy, NTT) approved as an adjunct therapy for patients with recurrent glioblastoma who are also receiving radiotherapy (101, 102) (ANCHIALE, NCT06271421). Nonetheless, nanoparticle-based delivery remains in the early pilot stage, requiring further research and documentation prior to approval. The limited availability of nano-delivery treatments stems from lengthy testing requirements, lack of standardized nanotoxicology assays, and high manufacturing costs (93). Moreover, in glioblastoma PDT is still at the stage of preclinical *in vitro* experimental phase (103). One example is the INDYGO trial; a prospective, non-randomized, single-center, open-label, phase I study (NCT 03048240) that reports safety and efficacy after intraoperative treatment of glioma with photodynamic therapy (PDT) after administration of 5-ALA acid (5 aminolevulinic acid hydrochloride) (98, 99). Another ongoing study in Germany is evaluating stereotactic biopsy followed by 5-ALA-based stereotactic PDT and the feasibility of 5-ALA in stereotactic interstitial PDT in a subset of adult glioma patients (103). 5-ALA is the only fluorescence-guided glioma surgery agent approved by the FDA (79).

## 6 Conclusions

This brief review presents a comparative analysis of various chemotherapeutic strategies for GBM treatment based on nanotechnology, providing insights into the relative effectiveness and potential of different NP systems. Indeed, recent advancements in NPs development are promising, given the complexity of the BBB microenvironment, and enabling a more efficient targeted drug delivery. Chemotherapeutic multifunctional NPs that combine imaging, targeting, and therapeutic capabilities hold significant promise in improving GBM outcomes. Nonetheless, clinical adoption of chemotherapeutic NPs for glioblastoma treatment is still in its early stages. Both research challenges and processing standardization issues are to be overcome to proceed toward a clinical practice. In the future, collaborative efforts among material science researchers and clinicians will be crucial to fully exploit the potential of chemotherapeutic NPs for glioblastoma.

## References

[1] Stupp R. Effect of tumor-treating fields plus maintenance temozolomide vs maintenance temozolomide alone on survival in patients with glioblastoma: A randomized clinical trial. JAMA. (2017) 318:2306–16. doi: 10.1001/jama.2017.18718, PMID: 29260225 PMC5820703

[2] Weller M. Challenges in the diagnoses and treatment of CNS tumors. Neurooncol Pract. (2019) 6:329. doi: 10.1093/nop/npz044, PMID: 31555446 PMC6753350

[3] Louis DN Perry A Wesseling P Brat DJ Cree IA Figarella- Branger D. The 2021 WHO classification of tumors of the central nervous system: a summary. Neuro Oncol. (2021) 23:1231–51. doi: 10.1093/neuonc/noab106, PMID: 34185076 PMC8328013

[4] Steeg PS. The blood-tumour barrier in cancer biology and therapy. Nat Rev Clin Oncol. (2021) 18:696–714. doi: 10.1038/s41571-021-00529-6, PMID: 34253912

[5] Noorani I de la Rosa J. Breaking barriers for glioblastoma with a path to enhanced drug delivery. Nat Commun. (2023) 14:5909. doi: 10.1038/s41467-023-41694-9, PMID: 37737212 PMC10517119

[6] Mathur R Wang Q Schupp PG Nikolic A Hilz S Hong C. Glioblastoma evolution and heterogeneity from a 3D whole-tumor perspective. Cell. (2024) 187:446–63.e16. doi: 10.1016/j.cell.2023.12.013, PMID: 38242087 PMC10832360

[7] Verdugo E Puerto I Medina MÁ. An update on the molecular biology of glioblastoma, with clinical implications and progress in its treatment. Cancer Commun (Lond). (2022) 42:1083–111. doi: 10.1002/cac2.12361, PMID: 36129048 PMC9648390

[8] Dewdney B Jenkins MR Best SA Freytag S Prasad K Holst J. From signalling pathways to targeted therapies: unravelling glioblastoma’s secrets and harnessing two decades of progress. Sig Transduct Target Ther. (2023) 8:400. doi: 10.1038/s41392-023-01637-8, PMID: 37857607 PMC10587102

[9] Yang K Wu Z Zhang H Zhang N Wu W Wang Z. Glioma targeted therapy: insight into future of molecular approaches. Mol Cancer. (2022) 21:39. doi: 10.1186/s12943-022-01513-z, PMID: 35135556 PMC8822752

[10] Chan HY Choi J Jackson C Lim M. Combination immunotherapy strategies for glioblastoma. J Neurooncol. (2021) 151:375–91. doi: 10.1007/s11060-020-03481-0, PMID: 33611705

[11] Hasan I Roy S Ehexige E Wu R Chen Y Gao Z. A state-of-the-art liposome technology for glioblastoma treatment. Nanoscale. (2023) 15:18108–38. doi: 10.1039/d3nr04241c, PMID: 37937394

[12] Wiley DT Webster P Gale A Davis ME. Transcytosis and brain uptake of transferrin-containing nanoparticles by tuning avidity to transferrin receptor. Proc Natl Acad Sci U.S.A. (2013) 110:8662–7. doi: 10.1073/pnas.1307152110, PMID: 23650374 PMC3666717

[13] Tian X Leite DM Scarpa E Nyberg S Fullstone G Forth J. On the shuttling across the blood-brain barrier via tubule formation: mechanism and cargo avidity bias. Sci Adv. (2020) 6:eabc4397. doi: 10.1126/sciadv.abc4397, PMID: 33246953 PMC7695481

[14] Anraku Y Kuwahara H Fukusato Y Mizoguchi A Ishii T Nitta K. Glycaemic control boosts glucosylated nanocarrier crossing the BBB into the brain. Nat Commun. (2017) 8:1001. doi: 10.1038/s41467-017-00952-3, PMID: 29042554 PMC5645389

[15] Roberts MJ Bentley MD Harris JM. Chemistry for peptide and protein pegylation. Adv Drug Deliv Rev. (2002) 54:459–76. doi: 10.1016/s0169-409x(02)00022-4, PMID: 12052709

[16] Ghaznavi H Afzalipour R Khoei S Sargazi S Shirvalilou S Sheervalilou R. New insights into targeted therapy of glioblastoma using smart nanoparticles. Cancer Cell Int. (2024) 24:160. doi: 10.1186/s12935-024-03331-3, PMID: 38715021 PMC11077767

[17] Wadajkar AS Dancy JG Hersh DS Anastasiadis P Tran NL Woodworth GF. Tumor-targeted nanotherapeutics: overcoming treatment barriers for glioblastoma. Wiley Interdiscip Rev Nanomed Nanobiotechnol. (2017) 9:10.1002/wnan.1439. doi: 10.1002/wnan.1439, PMID: 27813323 PMC5418115

[18] Gusmão LA Matsuo FS Barbosa HF Tedesco AC. Advances in nano-based materials for glioblastoma multiforme diagnosis: A mini-review. Front Nanotechnol. (2022) 4:836802. doi: 10.3389/fnano.2022.836802

[19] Zhang H Wang T Liu H Ren F Qiu W Sun Q. Second near-infrared photodynamic therapy and chemotherapy of orthotopic Malignant glioblastoma with ultra-small Cu2-xSe nanoparticles. Nanoscale. (2019) 11:7600–8. doi: 10.1039/c9nr01789e, PMID: 30968107

[20] Lu VM Jue TR McDonald KL. Cytotoxic lanthanum oxide nanoparticles sensitize glioblastoma cells to radiation therapy and temozolomide: an *in vitro* rationale for translational studies. Sci Rep. (2020) 10:18156. doi: 10.1038/s41598-020-75372-3, PMID: 33097778 PMC7584621

[21] Zhan W. Delivery of liposome encapsulated temozolomide to brain tumour: Understanding the drug transport for optimisation. Int J Pharm. (2019) 557:280–92. doi: 10.1016/j.ijpharm.2018.12.065, PMID: 30599226

[22] Saha S Yakati V Shankar G Jaggarapu MMCS Moku G MadhuSudana K. Amphetamine decorated cationic lipid nanoparticles cross the blood-brain barrier: therapeutic promise for combating glioblastoma. J Mater Chem B. (2020) 8:4318–30. doi: 10.1039/c9tb02700a, PMID: 32330214

[23] Malinovskaya Y Melnikov P Baklaushev V Gabashvili A Osipova N Mantrov S. Delivery of doxorubicin-loaded PLGA nanoparticles into U87 human glioblastoma cells. Int J Pharm. (2017) 524:77–90. doi: 10.1016/j.ijpharm.2017.03.049, PMID: 28359811

[24] Ahmed T Liu FF He C Abbasi AZ Cai P Rauth AM. Optimizing the design of blood-brain barrier-penetrating polymer-lipid-hybrid nanoparticles for delivering anticancer drugs to glioblastoma. Pharm Res. (2021) 38:1897–914. doi: 10.1007/s11095-021-03122-9, PMID: 34655006

[25] Ebrahimi Shahmabadi H Movahedi F Koohi Moftakhari Esfahani M Alavi SE Eslamifar A Mohammadi Anaraki G. Efficacy of Cisplatin-loaded polybutyl cyanoacrylate nanoparticles on the glioblastoma. Tumour Biol. (2014) 35:4799–806. doi: 10.1007/s13277-014-1630-9, PMID: 24443270

[26] Chiani M Toofani Milani A Nemati M Rezaeidian J Ehsanbakhsh H Ahmadi Z. Anticancer effect of cisplatin- loaded poly (Butylcyanoacrylate) nanoparticles on A172 brain cancer cells line. Asian Pac J Cancer Prev. (2019) 20:303–9. doi: 10.31557/APJCP.2019.20.1.303, PMID: 30678454 PMC6485583

[27] Maksimenko O Malinovskaya J Shipulo E Osipova N Razzhivina V Arantseva D. Doxorubicin-loaded PLGA nanoparticles for the chemotherapy of glioblastoma: Towards the pharmaceutical development. Int J Pharm. (2019) 572:118733. doi: 10.1016/j.ijpharm.2019.118733, PMID: 31689481

[28] Madani F Esnaashari SS Bergonzi MC Webster TJ Younes HM Khosravani M. Paclitaxel/methotrexate co-loaded PLGA nanoparticles in glioblastoma treatment: Formulation development and *in vitro* antitumor activity evaluation. Life Sci. (2020) 256:117943. doi: 10.1016/j.lfs.2020.117943, PMID: 32531377

[29] Madani F Morovvati H Webster TJ Najaf Asaadi S Rezayat SM Hadjighassem M. Combination chemotherapy via poloxamer 188 surface-modified PLGA nanoparticles that traverse the blood-brain-barrier in a glioblastoma model. Sci Rep. (2024) 14:19516. doi: 10.1038/s41598-024-69888-1, PMID: 39174603 PMC11341868

[30] Maleki H Hosseini Najafabadi MR Webster TJ Hadjighassem MR Sadroddiny E. Effect of Paclitaxel/etoposide co-loaded polymeric nanoparticles on tumor size and survival rate in a rat model of glioblastoma. Int J Pharm. (2021) 604:120722. doi: 10.1016/j.ijpharm.2021.120722, PMID: 34022255

[31] Caban-Toktas S Sahin A Lule S Esendagli G Vural I Karlı Oguz K. Combination of Paclitaxel and R-flurbiprofen loaded PLGA nanoparticles suppresses glioblastoma growth on systemic administration. Int J Pharm. (2020) 578:119076. doi: 10.1016/j.ijpharm.2020.119076, PMID: 31988035

[32] Arshad A Yang B Bienemann AS Barua NU Wyatt MJ Woolley M. Convection-enhanced delivery of carboplatin PLGA nanoparticles for the treatment of glioblastoma. PloS One. (2015) 10:e0132266. doi: 10.1371/journal.pone.0132266, PMID: 26186224 PMC4506141

[33] Xu Y Shen M Li Y Sun Y Teng Y Wang Y. The synergic antitumor effects of paclitaxel and temozolomide co-loaded in mPEG-PLGA nanoparticles on glioblastoma cells. Oncotarget. (2016) 7:20890–901. doi: 10.18632/oncotarget.7896, PMID: 26956046 PMC4991499

[34] Plichta Z Horák D Mareková D Turnovcová K Kaiser R Jendelová P. Poly[N-(2-hydroxy propyl) methacrylamide]-Modified Magnetic γ-F2 O3 Nanoparticles Conjugated with Doxorubicin for Glioblastoma Treatment. ChemMedChem. (2020) 15:96–104. doi: 10.1002/cmdc.201900564, PMID: 31670889

[35] Akyuva Y Nazıroğlu M. Silver nanoparticles potentiate antitumor and oxidant actions of cisplatin via the stimulation of TRPM2 channel in glioblastoma tumor cells. Chem Biol Interact. (2023) 369:110261. doi: 10.1016/j.cbi.2022.110261, PMID: 36403784

[36] Erthal LCS Shi Y Sweeney KJ Gobbo OL Ruiz-Hernandez E. Nanocomposite formulation for a sustained release of free drug and drug-loaded responsive nanoparticles: an approach for a local therapy of glioblastoma multiforme. Sci Rep. (2023) 13:5094. doi: 10.1038/s41598-023-32257-5, PMID: 36991081 PMC10060267

[37] Zhang Y Fu X Jia J Wikerholmen T Xi K Kong Y. Glioblastoma therapy using codelivery of cisplatin and glutathione peroxidase targeting siRNA from iron oxide nanoparticles. ACS Appl Mater Interfaces. (2020) 12:43408–21. doi: 10.1021/acsami.0c12042, PMID: 32885649

[38] Li Y Zhao Q Zhu X Zhou L Song P Liu B. Self-Assembled nanoparticles of natural bioactive molecules enhance the delivery and efficacy of paclitaxel in glioblastoma. CNS Neurosci Ther. (2024) 30:e14528. doi: 10.1111/cns.14528, PMID: 38044793 PMC11017454

[39] Zou Y Wang Y Xu S Liu Y Yin J Lovejoy DB. Brain co-delivery of temozolomide and cisplatin for combinatorial glioblastoma chemotherapy. Adv Mater. (2022) 34:e2203958. doi: 10.1002/adma.202203958, PMID: 35738390

[40] Mao J Meng X Zhao C Yang Y Liu G. Development of transferrin-modified poly(lactic-co-glycolic acid) nanoparticles for glioma therapy. Anticancer Drugs. (2019) 30:604–10. doi: 10.1097/CAD.0000000000000754, PMID: 30855310

[41] Wang L Liu C Qiao F Li M Xin H Chen N. Analysis of the cytotoxic effects, cellular uptake and cellular distribution of paclitaxel-loaded nanoparticles in glioblastoma cells *in vitro* . Exp Ther Med. (2021) 21:292. doi: 10.3892/etm.2021.9723, PMID: 33717235 PMC7885080

[42] Luo M Lewik G Ratcliffe JC Choi CHJ Mäkilä E Tong WY. Systematic evaluation of transferrin-modified porous silicon nanoparticles for targeted delivery of doxorubicin to glioblastoma. ACS Appl Mater Interfaces. (2019) 11:33637–49. doi: 10.1021/acsami.9b10787, PMID: 31433156

[43] Banstola A Duwa R Emami F Jeong JH Yook S. Enhanced caspase-mediated abrogation of autophagy by temozolomide-loaded and panitumumab-conjugated poly (lactic-co-glycolic acid) nanoparticles in epidermal growth factor receptor overexpressing glioblastoma cells. Mol Pharm. (2020) 17:4386–400. doi: 10.1021/acs.molpharmaceut.0c00856, PMID: 33079558

[44] Wang L Wang X Shen L Alrobaian M Panda SK Almasmoum HA. Paclitaxel and naringenin-loaded solid lipid nanoparticles surface modified with cyclic peptides with improved tumor targeting ability in glioblastoma multiforme. BioMed Pharmacother. (2021) 138:111461. doi: 10.1016/j.biopha.2021.111461, PMID: 33706131

[45] Ullah I Chung K Bae S Li Y Kim C Choi B. Nose-to-brain delivery of cancer-targeting paclitaxel- loaded nanoparticles potentiates antitumor effects in Malignant glioblastoma. Mol Pharm. (2020) 17:1193–204. doi: 10.1021/acs.molpharmaceut.9b01215, PMID: 31944768

[46] Chung K Ullah I Kim N Lim J Shin J Lee SC. Intranasal delivery of cancer-targeting doxorubicin-loaded PLGA nanoparticles arrests glioblastoma growth. J Drug Target. (2020) 28:617–26. doi: 10.1080/1061186X.2019.1706095, PMID: 31852284

[47] Wang L Tang S Yu Y Lv Y Wang A Yan X. Intranasal delivery of temozolomide-conjugated gold nanoparticles functionalized with anti-ephA3 for glioblastoma targeting. Mol Pharm. (2021) 18:915–27. doi: 10.1021/acs.molpharmaceut.0c00911, PMID: 33417456

[48] Wang S Yu Y Wang A Duan X Sun Y Wang L. Temozolomide hexadecyl ester targeted PLGA nanoparticles for drug-resistant glioblastoma therapy via intranasal administration. Front Pharmacol. (2022) 13:965789. doi: 10.3389/fphar.2022.965789, PMID: 36059989 PMC9429944

[49] Chen H Wen J. Iron oxide nanoparticles loaded with paclitaxel inhibits glioblastoma by enhancing autophagy-dependent ferroptosis pathway. Eur J Pharmacol. (2022) 921:174860. doi: 10.1016/j.ejphar.2022.174860, PMID: 35278406

[50] Zhang J Sun Y Tian B Li K Wang L Liang Y. Multifunctional mesoporous silica nanoparticles modified with tumor-shedable hyaluronic acid as carriers for doxorubicin. Colloids Surf B Biointerfaces. (2016) 144:293–302. doi: 10.1016/j.colsurfb.2016.04.015, PMID: 27107383

[51] Deshmukh V Narwade M Gajbhiye KR. Intranasal delivery of paclitaxel-loaded ligand conjugated polymeric nanoparticles for targeted brain delivery. AAPS PharmSciTech. (2025) 26:49. doi: 10.1208/s12249-025-03046-2, PMID: 39900701

[52] Janjua TI Cao Y Ahmed-Cox A Raza A Moniruzzaman M Akhter DT. Efficient delivery of Temozolomide using ultrasmall large-pore silica nanoparticles for glioblastoma. J Control Release. (2023) 357:161–74. doi: 10.1016/j.jconrel.2023.03.040, PMID: 36965857

[53] Norouzi M Yathindranath V Thliveris JA Kopec BM Siahaan TJ Miller DW. Doxorubicin-loaded iron oxide nanoparticles for glioblastoma therapy: a combinational approach for enhanced delivery of nanoparticles. Sci Rep. (2020) 10:11292. doi: 10.1038/s41598-020-68017-y, PMID: 32647151 PMC7347880

[54] Kwon YM Je JY Cha SH Oh Y Cho WH. Synergistic combination of chemo−phototherapy based on temozolomide/ICG−loaded iron oxide nanoparticles for brain cancer treatment. Oncol Rep. (2019) 42:1709–24. doi: 10.3892/or.2019.7289, PMID: 31436296 PMC6775808

[55] Beola L Iturrioz-Rodríguez N Pucci C Bertorelli R Ciofani G. Drug-loaded lipid magnetic nanoparticles for combined local hyperthermia and chemotherapy against glioblastoma multiforme. ACS Nano. (2023) 17:18441–55. doi: 10.1021/acsnano.3c06085, PMID: 37698887 PMC10540267

[56] Lajous H Riva R Lelièvre B Tétaud C Avril S Hindré F. Hybrid Gd3+/cisplatin cross-linked polymer nanoparticles enhance platinum accumulation and formation of DNA adducts in glioblastoma cell lines. Biomater Sci. (2018) 6:2386–409. doi: 10.1039/c8bm00346g, PMID: 30023990

[57] Pulvirenti L Monforte F Lo Presti F Li Volti G Carota G Sinatra F. Synthesis of MIL-modified fe3O4 magnetic nanoparticles for enhancing uptake and efficiency of temozolomide in glioblastoma treatment. Int J Mol Sci. (2022) 23:2874. doi: 10.3390/ijms23052874, PMID: 35270016 PMC8911361

[58] Mao X Calero-Pérez P Montpeyó D Bruna J Yuste VJ Candiota AP. Intranasal administration of catechol-based pt(IV) coordination polymer nanoparticles for glioblastoma therapy. Nanomater (Basel). (2022) 12:1221. doi: 10.3390/nano12071221, PMID: 35407338 PMC9003391

[59] Neshastehriz A Khateri M Ghaznavi H Shakeri-Zadeh A. Investigating the therapeutic effects of alginate nanogel co-loaded with gold nanoparticles and cisplatin on U87-MG human glioblastoma cells. Anticancer Agents Med Chem. (2018) 18:882–90. doi: 10.2174/1871520618666180131112914, PMID: 29384064

[60] Jing Z Li M Wang H Yang Z Zhou S Ma J. Gallic acid-gold nanoparticles enhance radiation-induced cell death of human glioma U251 cells. IUBMB Life. (2021) 73:398–407. doi: 10.1002/iub.2436, PMID: 33372372 PMC7898864

[61] He C Zhang Z Ding Y Xue K Wang X Yang R. LRP1-mediated pH-sensitive polymersomes facilitate combination therapy of glioblastoma *in vitro* and *in vivo* . J Nanobiotechnol. (2021) 19:29. doi: 10.1186/s12951-020-00751-x, PMID: 33482822 PMC7821499

[62] Depciuch J Miszczyk J Maximenko A Zielinski PM Rawoj´c K Panek A. Gold nanopeanuts as prospective support for cisplatin in glioblastoma nano-chemo-radiotherapy. Int J Mol Sci. (2020) 21:9082. doi: 10.3390/ijms21239082, PMID: 33260340 PMC7730046

[63] Ganipineni LP Ucakar B Joudiou N Riva R Jérôme C Gallez B. Paclitaxel-loaded multifunctional nanoparticles for the targeted treatment of glioblastoma. J Drug Target. (2019) 27:614–23. doi: 10.1080/1061186X.2019.1567738, PMID: 30633585

[64] Zhu X Huang H Zhang Y Zhang H Hou L Zhang Z. Cit/CuS@Fe3O4-based and enzyme-responsive magnetic nanoparticles for tumor chemotherapy, photothermal, and photodynamic therapy. J Biomater Appl. (2017) 31:1010–25. doi: 10.1177/0885328216676159, PMID: 28178904

[65] Hersh AM Alomari S Tyler BM. Crossing the blood-brain barrier: advances in nanoparticle technology for drug delivery in neuro-oncology. Int J Mol Sci. (2022) 23:4153. doi: 10.3390/ijms23084153, PMID: 35456971 PMC9032478

[66] Öztürk K Kaplan M Çalış S. Effects of nanoparticle size, shape, and zeta potential on drug delivery. Int J Pharm. (2024) 666:124799. doi: 10.1016/j.ijpharm.2024.124799, PMID: 39369767

[67] Stevens MF Hickman JA Langdon SP Chubb D Vickers L Stone R. Antitumor activity and pharmacokinetics in mice of 8-carbamoyl-3-methyl-imidazo[5,1-d]-1,2,3,5-tetrazin-4(3H)-one (CCRG 81045; M & B 39831), a novel drug with potential as an alternative to dacarbazine. Cancer Res. (1987) 47:5846–52., PMID: 3664486

[68] Stupp R Mason WP van den Bent MJ Weller M Fisher B Taphoorn MJB. Radiotherapy plus concomitant and adjuvant temozolomide for glioblastoma. N Engl J Med. (2005) 352:987–96. doi: 10.1056/nejmoa043330, PMID: 15758009

[69] van den Bent MJ Hegi ME Stupp R. Recent developments in the use of chemotherapy in brain tumours. Eur J Cancer. (2006) 42:582–8. doi: 10.1016/j.ejca.2005.06.031, PMID: 16427778

[70] Dutra JAP Luiz MT Tavares Junior AG Di Filippo LD Carvalho SG Chorilli M. Temozolomide: an overview of biological properties, drug delivery nanosystems, and analytical methods. Curr Pharm Des. (2022) 28:2073–88. doi: 10.2174/1381612828666220603152918, PMID: 35658888

[71] Ananta JS Paulmurugan R Massoud TF. Temozolomide-loaded PLGA nanoparticles to treat glioblastoma cells: a biophysical and cell culture evaluation. Neurol Res. (2016) 38:51–9. doi: 10.1080/01616412.2015.1133025, PMID: 26905383

[72] Ramalho MJ Torres ID Loureiro JA Lima J Pereira MC. Transferrin-conjugated PLGA nanoparticles for co-delivery of temozolomide and bortezomib to glioblastoma cells. ACS Appl Nano Mater. (2023) 6:14191–203. doi: 10.1021/acsanm.3c02122, PMID: 37588263 PMC10426337

[73] Sharifi-Rad J Quispe C Patra JK Singh YD Panda MK Das G. Paclitaxel: application in modern oncology and nanomedicine-based cancer therapy. Oxid Med Cell Longev. (2021) 2021:3687700. doi: 10.1155/2021/3687700, PMID: 34707776 PMC8545549

[74] Cai Q Li X Xiong H Fan H Gao X Vemireddy V. Optical blood-brain-tumor barrier modulation expands therapeutic options for glioblastoma treatment. Nat Commun. (2023) 14:4934. doi: 10.1038/s41467-023-40579-1, PMID: 37582846 PMC10427669

[75] Rottenberg S Disler C Perego P. The rediscovery of platinum-based cancer therapy. Nat Rev Cancer. (2021) 21:37–50. doi: 10.1038/s41568-020-00308-y, PMID: 33128031

[76] Aryal S Hu CM Fang RH Dehaini D Carpenter C Zhang DE. Erythrocyte membrane-cloaked polymeric nanoparticles for controlled drug loading and release. Nanomed (Lond). (2013) 8:1271–80. doi: 10.2217/nnm.12.153, PMID: 23409747

[77] Lin X Ma X Zhao S Yao J Han L Jing Y. Cardiovascular toxicity in antitumor therapy: biological and therapeutic insights. Trends Cancer. (2024) 10:920–34. doi: 10.1016/j.trecan.2024.07.004, PMID: 39097431

[78] Li G Wang C Jin B Sun T Sun K Wang S. Advances in smart nanotechnology-supported photodynamic therapy for cancer. Cell Death Discov. (2024) 10:466. doi: 10.1038/s41420-024-02236-4, PMID: 39528439 PMC11554787

[79] Liu Z Mela A Argenziano MG Banu MA Furnari J Kotidis C. Single-cell analysis of 5-aminolevulinic acid intraoperative labeling specificity for glioblastoma. J Neurosurg. (2023) 140:968–78. doi: 10.3171/2023.7.JNS23122, PMID: 37773782 PMC10535619

[80] Liu D Dai X Ye L Wang H Qian H Cheng H. Nanotechnology meets glioblastoma multiforme: Emerging therapeutic strategies. Wiley Interdiscip Rev Nanomed Nanobiotechnol. (2023) 15:e1838. doi: 10.1002/wnan.1838, PMID: 35959642

[81] Liu Z Wang J Qiu K Liao X Rees TW Ji L. Fabrication of Red blood cell membrane-camouflaged Cu2-xSe nanoparticles for phototherapy in the second near-infrared window. Chem Commun. (2019) 55:6523–6. doi: 10.1039/c9cc03148k, PMID: 31099806

[82] He Y Cong C He Y Hao Y Li C Wang S. Tumor hypoxia relief overcomes multidrug resistance and immune inhibition for self-enhanced photodynamic therapy. Chem Eng J. (2019) 375:122079. doi: 10.1016/j.cej.2019.122079

[83] Chen Q Feng L Liu J Zhu W Dong Z Wu Y. Intelligent albumin-mnO2 nanoparticles as pH-/H2 O2 -responsive dissociable nanocarriers to modulate tumor hypoxia for effective combination therapy. Adv Mater. (2016) 28:7129–36. doi: 10.1002/adma.201601902, PMID: 27283434

[84] Perini G Palmieri V Ciasca G D’Ascenzo M Gervasoni J Primiano A. Graphene quantum dots’ Surface chemistry modulates the sensitivity of glioblastoma cells to chemotherapeutics. Int J Mol Sci. (2020) 21:6301. doi: 10.3390/ijms21176301, PMID: 32878114 PMC7503375

[85] Perini G Palmieri V Friggeri G Augello A De Spirito M Papi M. Carboxylated graphene quantum dots-mediated photothermal therapy enhances drug-membrane permeability, ROS production, and the immune system recruitment on 3D glioblastoma models. Cancer Nano. (2023) 14:13. doi: 10.1186/s12645-023-00168-9

[86] Zhou XQ Meng LB Huang Q Li J Zheng K Zhang FL. Synthesis and *in vitro* anticancer activity of Zinc (II) phthalocyanines conjugated with coumarin derivatives for dual photodynamic and chemotherapy. ChemMedChem. (2015) 10:304–11. doi: 10.1002/cmdc.201402401, PMID: 25369981

[87] Xiang H Chen H Tham HP Phua SZF Liu JG Zhao Y. Cyclometalated iridium (III)-complex-based micelles for glutathione-responsive targeted chemotherapy and photodynamic therapy. ACS Appl Mater Interfaces. (2017) 9:27553–62. doi: 10.1021/acsami.7b09506, PMID: 28749655

[88] Srinivasan S Bhardwaj V Nagasetti A Fernandez-Fernandez A McGoron AJ. Multifunctional surface-enhanced raman spectroscopy-detectable silver nanoparticles for combined photodynamic therapy and PH-triggered chemotherapy. J Biomed Nanotechnol. (2016) 12:2202–19. doi: 10.1166/jbn.2016.2312, PMID: 29372971

[89] Yuan Y Min Y Hu Q Xing B Liu B. NIR photoregulated chemo-and photodynamic cancer therapy based on conjugated polyelectrolyte–drug conjugate encapsulated upconversion nanoparticles. Nanoscale. (2014) 6:11259–72. doi: 10.1039/c4nr03302g, PMID: 25130329

[90] Liu Sf Li MJ Liang B Sun W Shao Y Hu X. Breaking the barrier: Nanoparticle-enhanced radiotherapy as the new vanguard in brain tumor treatment. Front Pharmacol. (2024) 15:1394816. doi: 10.3389/fphar.2024.1394816, PMID: 39021831 PMC11252536

[91] Chen K Chang C Liu Z Zhou Y Xu Q Li C. Hyaluronic acid targeted and pH-responsive nanocarriers based on hollow mesoporous silica nanoparticles for chemo-photodynamic combination therapy. Colloids Surf B Biointerfaces. (2020) 194:111166. doi: 10.1016/j.colsurfb.2020.111166, PMID: 32521461

[92] Meng X Wang K Lv L Zhao Y Sun C Ma L. Photothermal/photodynamic therapy with immune-adjuvant liposomal complexes for effective gastric cancer therapy. Part Part Syst Charact. (2019) 36:1900015. doi: 10.1002/ppsc.201900015

[93] Reddy S Tatiparti K Sau S Iyer AK. Recent advances in nano delivery systems for blood-brain barrier (BBB) penetration and targeting of brain tumors. Drug Discov Today. (2021) 26:1944– 52. doi: 10.1016/j.drudis.2021.04.008, PMID: 33865978

[94] Aldape K Brindle KM Chesler L Chopra R Gajjar A Gilbert MR. Challenges to curing primary brain tumours. Nat Rev Clin Oncol. (2019) 16:509–20. doi: 10.1038/s41571-019-0177-5, PMID: 30733593 PMC6650350

[95] Beier CP Schmid C Gorlia T Kleinletzenberger C Beier D Grauer O. RNOP-09: pegylated liposomal doxorubicine and prolonged temozolomide in addition to radiotherapy in newly diagnosed glioblastoma–a phase II study. BMC Cancer. (2009) 9:308. doi: 10.1186/1471-2407-9-308, PMID: 19725960 PMC2749868

[96] Huang H Feng W Chen Y Shi J. Inorganic nanoparticles in clinical trials and translations. Nano Today. (2020) 35:100972. doi: 10.1016/j.nantod.2020.100972

[97] Thivat E Casile M Moreau J Molnar I Dufort S Seddik K. Phase I/II study testing the combination of AGuIX nanoparticles with radiochemotherapy and concomitant temozolomide in patients with newly diagnosed glioblastoma (NANO-GBM trial protocol). BMC Cancer. (2023) 23:344. doi: 10.1186/s12885-023-10829-y, PMID: 37060055 PMC10105392

[98] Vermandel M Dupont C Lecomte F Leroy HA Tuleasca C Mordon S. Standardized intraoperative 5-ALA photodynamic therapy for newly diagnosed glioblastoma patients: a preliminary analysis of the INDYGO clinical trial. J Neurooncol. (2021) 152:501–14. doi: 10.1007/s11060-021-03718-6, PMID: 33743128

[99] Peciu-Florianu I Vannod-Michel Q Vauleon E Bonneterre ME Reyns N. Long term follow-up of patients with newly diagnosed glioblastoma treated by intraoperative photodynamic therapy: an update from the INDYGO trial (NCT03048240). J Neurooncol. (2024) 168:495–505. doi: 10.1007/s11060-024-04693-4, PMID: 38753093 PMC11186870

[100] Biau J Durando X Boux F Molnar I Moreau J Leyrat B. NANO-GBM trial of AGuIX nanoparticles with radiotherapy and temozolomide in the treatment of newly diagnosed Glioblastoma: Phase 1b outcomes and MRI-based biodistribution. Clin Transl Radiat Oncol. (2024) 48:100833. doi: 10.1016/j.ctro.2024.100833, PMID: 39184998 PMC11342789

[101] Maier-Hauff K Ulrich F Nestler D Niehoff H Wust P Thiesen B. Efficacy and safety of intratumoral thermotherapy using magnetic iron-oxide nanoparticles combined with external beam radiotherapy on patients with recurrent glioblastoma multiforme. J Neurooncol. (2011) 103:317–24. doi: 10.1007/s11060-010-0389-0, PMID: 20845061 PMC3097345

[102] Shirvalilou S Khoei S Esfahani AJ Kamali M Shirvaliloo M Sheervalilou R. Magnetic Hyperthermia as an adjuvant cancer therapy in combination with radiotherapy versus radiotherapy alone for recurrent/progressive glioblastoma: a systematic review. J Neurooncol. (2021) 152:419–28. doi: 10.1007/s11060-021-03729-3, PMID: 33713248

[103] Bhanja D Wilding H Baroz A Trifoi M Shenoy G Slagle-Webb B. Photodynamic therapy for glioblastoma: illuminating the path toward clinical applicability. Cancers (Basel). (2023) 15:3427. doi: 10.3390/cancers15133427, PMID: 37444537 PMC10341187

